# Stretch-Induced Spin-Cast Membranes Based on Semi-Crystalline Polymers for Efficient Microfiltration

**DOI:** 10.3390/polym16131799

**Published:** 2024-06-25

**Authors:** Junaid Saleem, Zubair Khalid Baig Moghal, Ahsan Hafeez, Samra Sajjad, Mohammad Shoaib, Johaina Alahmad, Gordon McKay

**Affiliations:** 1 Division of Sustainable Development, College of Science and Engineering, Hamad Bin Khalifa University, Qatar Foundation, Doha P.O. Box 5825, Qatar; 2 Center for Advanced Materials, Qatar University, Doha P.O. Box 2713, Qatar; 3 Department of Chemical Engineering, Qatar University, Doha P.O. Box 2713, Qatar

**Keywords:** spin-casting, plastic waste, microfiltration, membrane, stretching, strength, polyethylene

## Abstract

Microfiltration membranes derived from semi-crystalline polymers face various challenges when synthesized through the extrusion–casting technique, including the use of large quantities of polymer, long casting times, and the generation of substantial waste. This study focuses on synthesizing these membranes using spin-casting, followed by stretch-induced pore formation. Recycled high-density polyethylene (HDPE) and virgin polyethylene powder, combined with a calcium carbonate filler, were used as the source materials for the membranes. The influence of the polymer–filler ratio with and without stretching on the morphology, tensile strength, and water flow rate was investigated. Optimal conditions were determined, emphasizing a balance between pore structure and mechanical integrity. The permeable membrane exhibited a water flow rate of 19 mL/min, a tensile strength of 32 MPa, and a water contact angle of 126°. These membranes effectively eliminated suspended particles from water, with their performance evaluated against that of commercially available membranes. This research, carried out utilizing the spin-casting technique, outlines a synthesis route for microfiltration membranes tailored to semi-crystalline polymers and their plastic forms.

## 1. Introduction

Microfiltration membranes are widely utilized in water purification systems, surpassing traditional thermal-based separation methods in their effectiveness [1,2]. Characterized by pore sizes ranging from 0.1 to 10 µm, these membranes efficiently remove suspended particles and impurities, employing the physical size exclusion principle [3]. Moreover, they serve as a pre-filtration stage, preventing blockage and early membrane fouling, being particularly beneficial for expensive reverse osmosis membranes [4]. This approach not only ensures efficient separation but also minimizes energy consumption, making microfiltration a cost-effective solution for water treatment [5,6].

Electrospinning is a widely recognized process used for the continuous production of fibrous filtration membranes, with their fibers ranging from sub-micron-sized to nano-sized [3]. However, this technique is limited to the polymers that are soluble at room temperature and cannot be used for semi-crystalline polymers such as polyolefins, which include polypropylene (PP) and high-density polyethylene (HDPE) [3,7]. In addition, polyolefins are the major component of total plastic waste due to their extensive usage as commodity plastics [8,9,10,11,12]. These plastics are non-biodegradable and have the potential to cause environmental harm [13,14,15,16,17,18]. Hence, there should be a technique that caters towards semi-crystalline polymers and their recycled plastic forms.

An alternative method for synthesizing microfiltration membranes utilizing semi-crystalline polymers involves the extrusion–hot-pressing technique. In this process, a polymer is extruded alongside particulate matter or filler [19]. The resulting extruded gel undergoes subsequent hot-pressing or extrusion–casting to shape a thin sheet. This sheet is then subjected to hot-drawing, either uniaxially or biaxially, creating pores at the interface between the polymer and filler [19,20,21,22,23,24]. Widely adopted in commercial applications, this extrusion–stretching route has proven to be efficient and scalable [25,26,27,28,29]. Notably, polyethylene (PE) is among the most preferred polymers due to its favorable attributes, which include its low cost, processability, scalability, and versatility [23,30].

Despite its notable advantages, the extrusion–stretching route comes with inherent challenges. This method demands a substantial quantity of polymer, leading to prolonged casting times due to the involvement of multiple steps. Additionally, it imposes high energy requirements and generates a considerable amount of waste. In contrast, spin-casting emerges as a favorable technique for low-scale and laboratory setups, offering advantages such as reduced polymer requirements, shorter casting times, and minimized wastage.

In this study, our objective was to synthesize a microfiltration membrane based on semi-crystalline polymers or plastics, employing a unique combination of spin-casting, annealing, and stretch-induced pore formation. We present a microfiltration membrane synthesized from a blend combining recycled HDPE and virgin polyethylene powder in a ratio of 30:70 wt. %, along with the inclusion of calcium carbonate (CaCO_3_) as a filler. The membrane is specifically designed to efficiently remove suspended particles from water. Various polymer and filler combinations were examined, with and without stretching, and the optimum conditions were achieved based on pore uniformity and the overall strength of the membrane. The flow rate and mechanical strength values of the as-prepared membranes were compared with those of the commercial microfiltration membranes. The integration of stretch-induced thin-film membranes for semi-crystalline polymers using spin-casting provides an alternate method in the realm of microfiltration technology.

## 2. Experimental Section

### 2.1. Materials and Methods

*p*-cymene was used as a solvent and purchased from Njnq Bio-tech Ltd. (Beijing, China). Waste HDPE bottles were collected locally. Ultrahigh-molecular-weight polyethylene (UHMWPE) with a molecular weight of 3–6 million from Sigma (St. Louis, MO, USA) was used as received. CaCO_3_ was procured from Sigma and used as a filler without further purification. A customized glass plate with an area of 25 cm^2^ served as the solid substrate for membrane fabrication. The annealing of the thin membrane films was conducted on a Heidolph hot plate. Durapore 0.22 µm PVDF membrane and Cytiva Cellulose Acetate 0.45 µm were used as commercial membranes.

### 2.2. Formulation and Preparation of Polyethylene and Filler Composite Membranes

Preparation of M1 membrane: A total of 700 mg of UHMWPE and 300 mg of HDPE were weighed and placed in a round-bottomed flask. Subsequently, 100 mL of *p*-cymene was added to the flask and heated to 130–135 °C. The solution was stirred for 15–20 min or until complete dissolution of the polymers occurred. The resulting solution was termed as the M1 solution and then poured onto the glass substrate and allowed to spin. The spin coating process involved four steps: 300 rpm for 10 s (step one), 700 rpm for 30 s (step two), 1000 rpm for 60 s (step three), and 3000 rpm for 120 s (step four). Subsequently, the glass substrate with the polymer was detached from the chuck and placed on a hot plate at 130 °C for 2–5 min. The polymer membrane was then removed from the substrate by peeling, resulting in a polymer thin film. This thin film was loaded onto the uniaxial stretching machine and stretched at 120 °C after holding for 3 min, maintaining a stretching speed of 10 mm/min. The resulting film was unloaded and utilized as the M1 membrane. A brief summary of the process is provided below:

M1: A mixture of 700 mg UHMWPE and 300 mg HDPE in 100 mL *p*-cymene heated to 130–135 °C. Spin-coated in four steps, then stretched.

M2: Same process as M1 but with the addition of 100 mg filler, not stretched.

M3: Same as M2 but stretched after spin-coating.

M4: Similar to M3 but with 400 mg filler added.

M5: Similar to M4 with 700 mg filler.

M6: Similar to M5 with 1000 mg filler.

The procedure for each membrane followed a consistent pattern, with slight variations in filler quantity and stretching. Refer to Table 1 and Table 2 for a summary of the membranes, their compositions, and their stretching ratios.

### 2.3. Water Filtration Test

The polymer composite microfiltration membrane was placed on a filtration system, and mud water collected from a swamp was poured onto it. After 10 min, the filtrate was collected and checked for absorbance intensity before and after filtration. The filtration was conducted under suction, which meant it required significantly less time compared to using gravitational pressure. The pH of the mud water before filtration was 6.3, and the pH after filtration was 6.4.

### 2.4. Membrane Regeneration

The membranes were regenerated by backwashing with water for 1 min, followed by sonication in a water bath for 5 min to ensure the complete removal of any impurities.

## 3. Results and Discussion

### 3.1. Material Characterization and Integration of Filler

**Figure 1:**
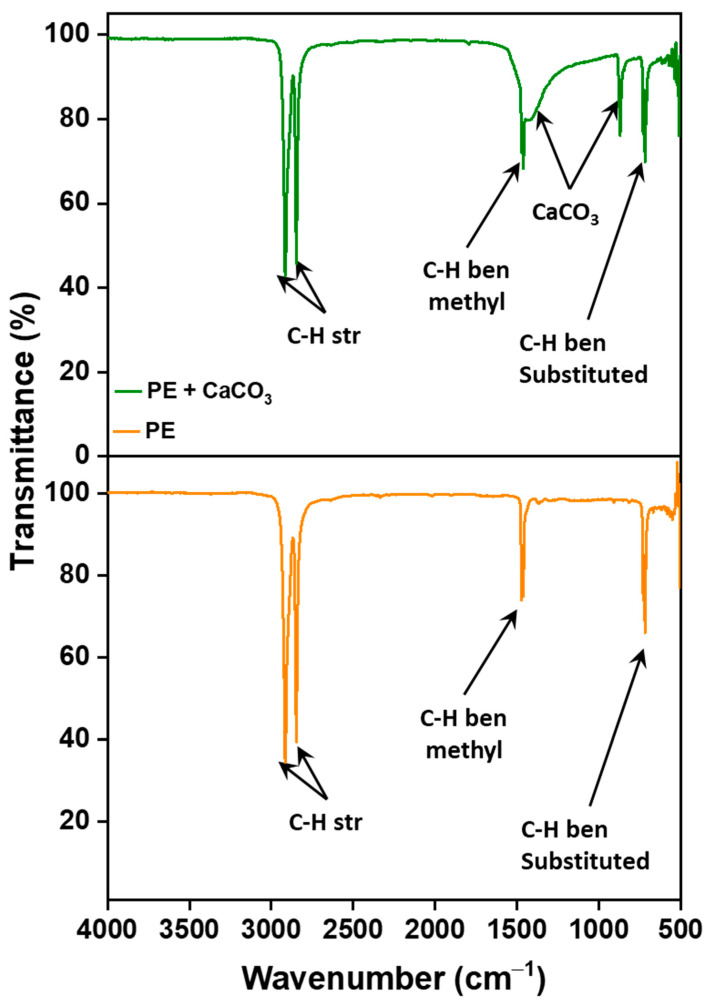
FTIR spectra showing functional group peaks for polymer–CaCO_3_ composite microfiltration membrane and pure PE.

The incorporation of the filler into the polymer matrix was verified through Fourier Transform Infrared Spectroscopy (FTIR). This confirmation was achieved by comparing the spectra of the composite membrane with those of pure polyethylene, as illustrated in Figure 1. The filler’s peaks are distinctly observed at around 1440 cm^−1^ (asymmetric CO stretching), 873–898 cm^−1^ (out-of-plane deformation of carbonate), and 712 cm^−1^ (OCO bending in-plane deformation vibrations of CaCO_3_), respectively, confirming the presence of filler and its successful integration with the polymer [31]. More importantly, the absence of new peaks in the FTIR spectrum suggests that there are no chemical changes occurring within the membrane. Instead, the interaction between the polymer and the filler is predominantly physical. This is supported by the observation that both PE and CaCO_3_ retain their characteristic peaks, signifying the preservation of their individual chemical identities within the composite structure.

**Figure 2:**
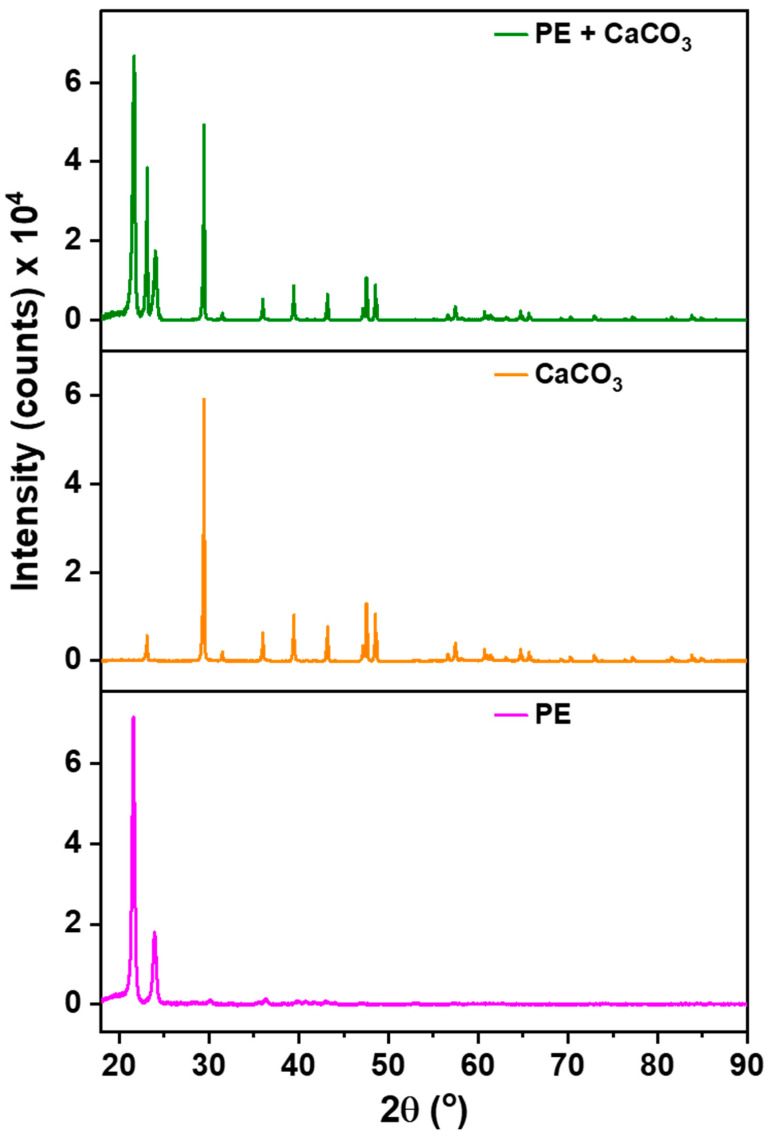
XRD patterns of polymer–filler composite membrane, pure CaCO_3_, and pure PE.

These results are further supported via our XRD analysis, Figure 2, which was conducted to check the presence of filler within the membrane’s structure. The significance of this examination arises from the potential release of filler during synthesis, especially given its significantly higher density compared to the polymer. The polymer (PE) exhibits characteristic peaks at 22° and 24°, serving as reference points. All other discernible peaks in the XRD spectrum are attributed to the filler.

### 3.2. Thermal Behavior of the Composite

**Figure 3:**
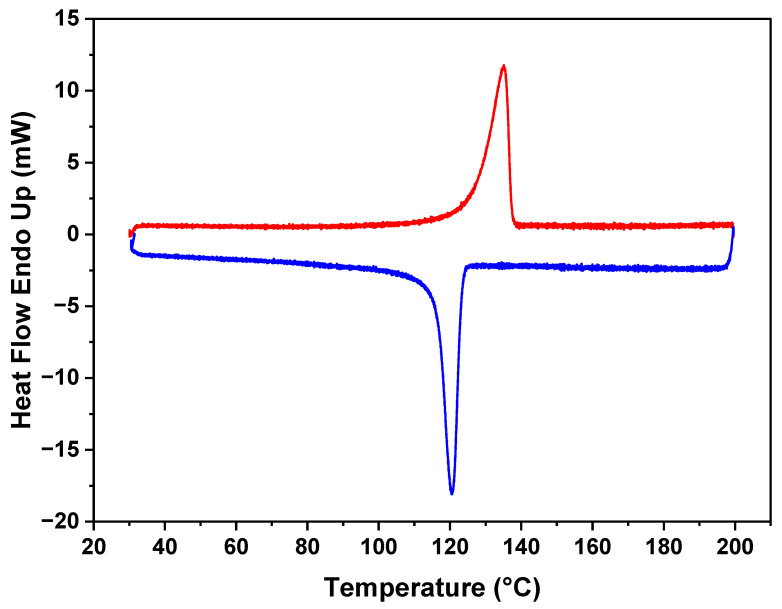
DSC plot of polymer–CaCO_3_ composite showing melting (red)and crystallization (blue) peaks.

The DSC results in Figure 3 reveal the distinct thermal characteristics of the polymer in the membrane. The figure indicates that the melting point for the polyethylene (PE) is 130 °C. Additionally, during the crystallization cooling phase, a melting point of 120 °C is observed, demonstrating the ability of the polymer to undergo reversible crystallization. Notably, the polymer maintains its melting behavior, and no discernible changes were observed in subsequent analyses. This stability in the thermal behavior of the polymer within the membrane demonstrates its resilience and the preservation of its intrinsic properties.

### 3.3. Mechanical Strength and Filler Content Influence

**Figure 4:**
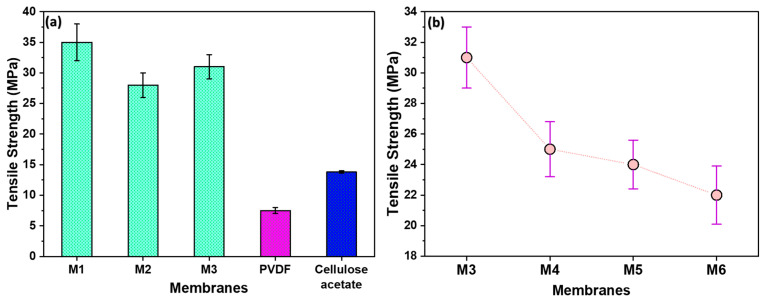
(**a**) The tensile strengths of the as-prepared membranes compared with those of commercial ones; (**b**) the tensile strengths of membranes prepared using filler, showing decrease in strength with increase in filler quantity.

In Figure 4a, the impact of filler and stretching on membrane strength was examined. M1, stretched without filler, exhibited a higher strength, suggesting that the absence of filler contributes to enhanced membrane robustness. On the contrary, M2, containing filler, demonstrated increased break points and a consequent reduction in strength. Notably, M3, stretched 1.8 times, exhibited comparatively higher strength than M2. This is due to the alignment of polymeric chains in the direction of stretching. The developed membranes had significantly higher tensile strengths than commercially available membranes such as Polyvinylidene Fluoride (PVDF) and Cellulose Acetate.

In Figure 4b, the influence of filler amount on membrane strength is shown. M3 has the highest strength, as it contains the lowest filler content in a polymer–filler ratio of 10:1. Conversely, M6, with the highest filler content (ratio of 10:10), exhibited the lowest strength due to the presence of more break points. Given its superior strength, M3 was selected for further characterizations and membrane performance tests.

### 3.4. Pore Structure and Water Flow Rate

**Figure 5:**
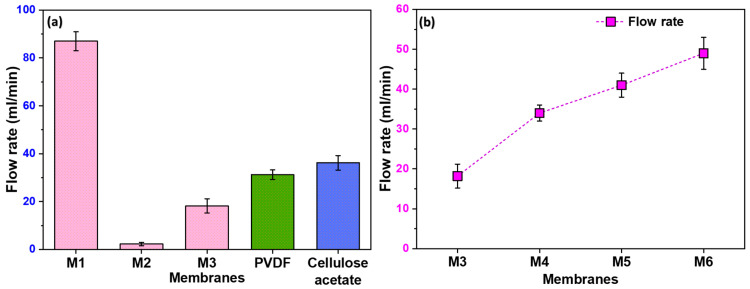
(**a**) The flow rate of distilled water through different membranes, including commercially available PVDF and Cellulose Acetate membranes; (**b**) the flow rates of membranes prepared using filler, showing increase in flow rate with increase in filler quantity.

Figure 5a illustrates the notable variations in the flow rate among the different membranes. M1 exhibited a high flow rate, attributed to its non-uniform pores resulting from the stretching process. The randomness of these pores, with some being notably larger than others, contributed to the enhanced flow rate. On the contrary, M2, lacking any formed pores, showed a negligible flow rate. Moreover, M3 exhibits a higher flow rate compared to M2 due to the uniformity in pore size and distribution. However, as anticipated, its flow rate is lower than that of M1. The observed trend highlights the importance of pore uniformity in filtration efficiency, with M3 striking a balance between the extremes of M1 and M2.

### 3.5. Influence of Filler Content on Flow Rate

In Figure 5b, we show the effect of filler content on flow rate, with membranes M4 to M6 demonstrating significantly higher flow rates than M3. This improvement in flow is directly linked to the formation of pores at the polymer–filler interface. Consequently, it becomes evident that an increase in filler content corresponds to higher pore formation, establishing a positive correlation between filler content and enhanced flow rates.

### 3.6. Surface Properties

**Figure 6:**
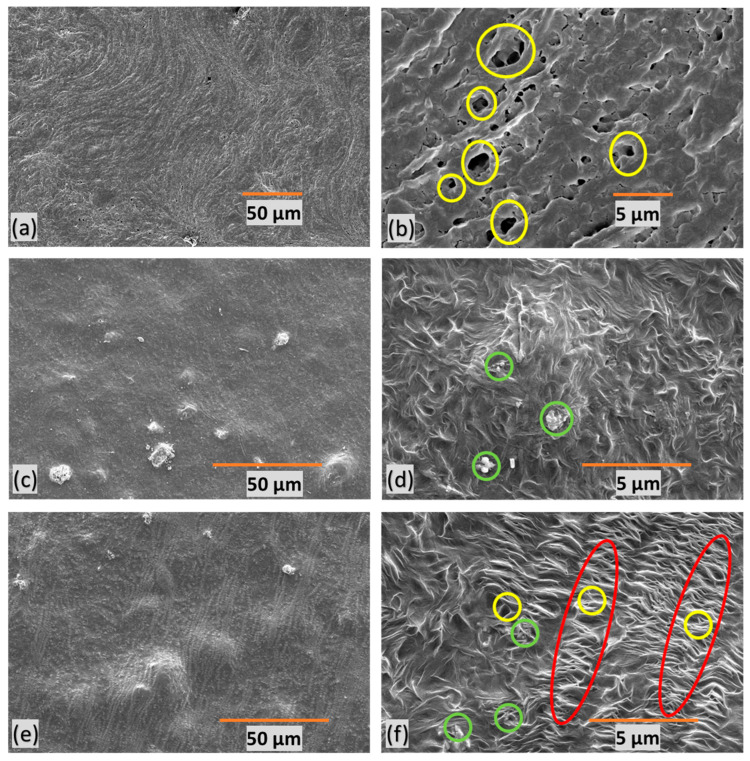
SEM images of polymer composite microfiltration membranes, the yellow circles represent pores, the green circles denote filler particles, and the red marks indicate stretched regions with micropores—(**a**,**b**) M1, (**c**,**d**) M2, (**e**,**f**), and M3—at different resolutions.

SEM images of polymer composite microfiltration membranes provide insights into their structural characteristics. In Figure 6, the yellow circles represent pores, the green circles denote filler particles, and the red marks indicate stretched regions with micropores. M1 (Figure 6a,b) exhibits undesirable features, characterized by poor orientation, non-uniformity, and a random distribution of large pores. Figure 6c,d present M2, revealing an absence of discernible pores due to the lack of stretching. This feature indicates the critical role stretching plays in pore formation within the membrane structure.

M3 (Figure 6e,f), on the other hand, displays a well-defined porous structure with a favorable orientation. This suggests that uniform stretching contributes to a more consistent and controlled pore size within the membrane. These insights into membrane morphology are crucial for understanding the impact of stretching, orientation, and filler content on pore formation, ultimately influencing water filtration efficiency.

**Figure 7:**
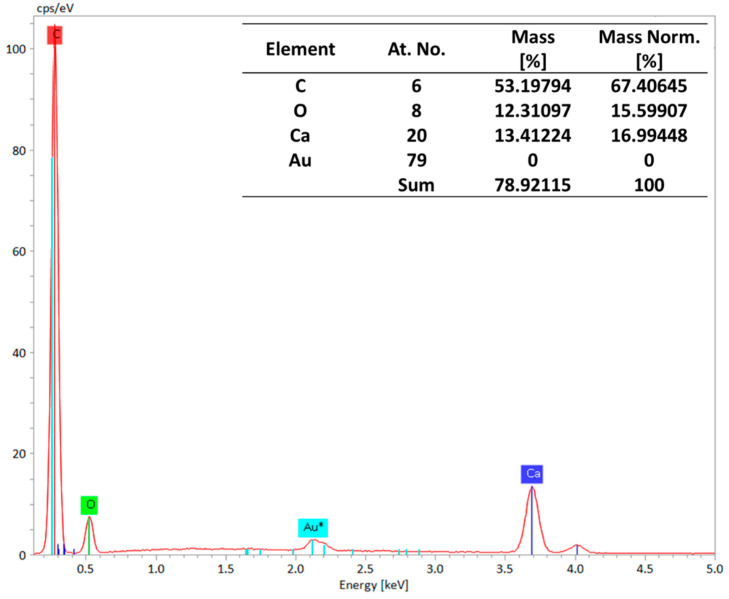
EDS spectra showing the presence of calcium and oxygen atoms, depicting the presence of CaCO_3_.

Our elemental analysis of the membranes was performed using Energy-Dispersive X-ray Spectroscopy (EDS), and the results are presented in Figure 7. EDS can detect a wide range of elements, from light elements (like carbon and oxygen) to heavy metals [32,33]. The EDS spectra confirm the presence of calcium and oxygen atoms, indicating that CaCO_3_ is present in the M3 membrane. These findings validate that CaCO_3_ was successfully incorporated into the membrane while retaining its overall structure. 

**Figure 8:**
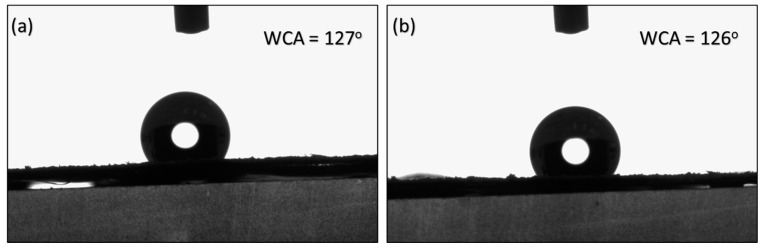
Water contact angles on (**a**) M2 and (**b**) M3 membranes.

The contact angles of M2 and M3 were recorded at 127° and 126°, respectively, as presented in Figure 8. The results suggest that stretching did not significantly influence the surface properties of the membrane. Instead, the contact angles appeared to be predominantly dependent on the synthesis method employed prior to stretching.

### 3.7. Filtration and Membrane Regeneration

**Figure 9:**
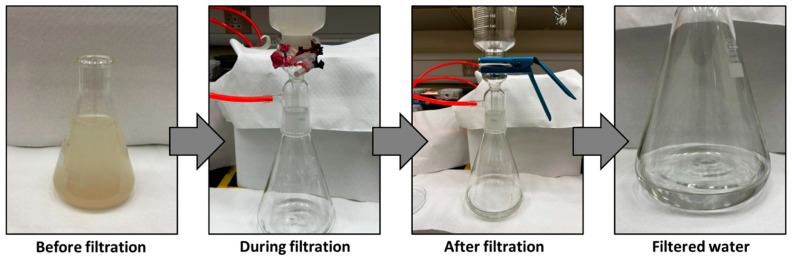
Mud water before and after filtration using M3 membrane.

**Figure 10:**
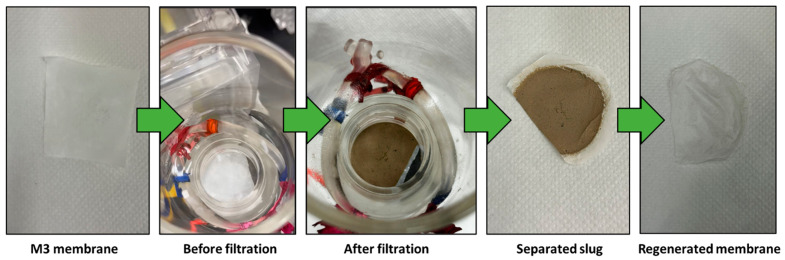
M3 membrane before and after filtration.

Figure 9 illustrates the outcomes of separating a mud water emulsion as a representative example of us using membrane M3. The filtered water obtained is transparent compared to the original brownish-white feed emulsion before filtration. Figure 10 provides insight into a water filtration process using mud water, presenting a comparison before and after membrane filtration. It is worth noting that the membranes possess the ability to undergo regeneration post-filtration, enabling their reuse across multiple cycles.

### 3.8. Filtration Efficiency Comparison and Recyclability

**Figure 11:**
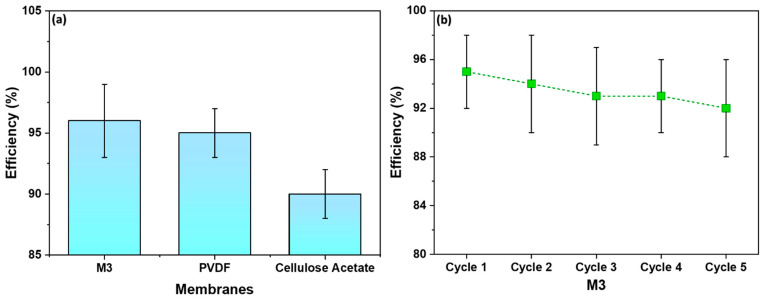
(**a**) A comparison of membrane efficiency with commercial membranes; (**b**) recyclability of M3 with respect to water filtration efficiency.

Figure 11a displays the efficiency of M3 in comparison to commercial membranes—PVDF and Cellulose Acetate. The M3 membrane showed a water filtration efficiency of 95%, slightly higher than the PVDF membrane, which demonstrated an efficiency of 93%. On the other hand, the Cellulose Acetate membrane illustrated the lowest efficiency—90%. Figure 11b shows the recyclability of M3 with respect to water filtration efficiency. The initial efficiency was 95% and after five cycles a similar efficiency was retained. As shown, M3 is recyclable for at least five cycles, with an efficiency of 92%.

The observed slightly higher filtration efficiency of the M3 membrane serves as validation of its well-defined porous structure. The uniform and controlled pores in the membrane, resulting from the combination of spin-casting and uniaxial stretching, play a pivotal role in blocking mud particles while facilitating the permeation of water. This outcome aligns with the SEM images presented earlier, highlighting the importance of pore uniformity in achieving enhanced filtration efficiency.

The synthesized microfiltration (MF) membrane offers several notable advantages: (a) The membranes exhibit a tensile strength of 32 MPa, which is significantly higher than that of commercially available membranes like Polyvinylidene Fluoride (PVDF) and Cellulose Acetate. This high mechanical strength ensures the membrane’s durability and robustness under operational conditions. (b) The use of spin-casting combined with stretch-induced pore formation is a more efficient fabrication method compared to traditional extrusion–casting techniques. This method offers several benefits in line with environmental management practices [34]. It reduces the quantity of polymer required, shortens casting times, and minimizes waste generation. The incorporation of recycled HDPE further enhances the environmental sustainability of the process. (c) The membrane achieved a high water flow rate of 19 mL/min, demonstrating efficient water permeability. The well-defined and uniform porous structure, achieved through controlled stretching, contributes to its high filtration efficiency. The membrane exhibits a water filtration efficiency of 95%, outperforming commercial alternatives like PVDF (93%) and Cellulose Acetate (90%). (d) The combination of polymer and filler, along with precise stretching techniques, results in a membrane with a favorable and uniform pore structure. This uniformity is crucial for effective filtration, ensuring consistent separation performance across the membrane surface. (e) The membrane can be regenerated and reused across multiple filtration cycles, maintaining an efficiency of 92% after five cycles. This recyclability aspect highlights the membrane’s long-term usability and cost-effectiveness for water treatment applications. (f) Lastly, the utilization of recycled HDPE as a primary material for membrane synthesis not only addresses the issue of plastic waste but also promotes sustainable manufacturing practices.

The superior filtration efficiency of the M3 membrane suggests its potential for practical applications in microfiltration technology. Its ability to outperform commercial membranes, coupled with the advantages of reduced polymer requirements and the shorter casting times associated with the spin-casting technique, positions the M3 membrane as a promising candidate for water treatment and purification processes.

## 4. Conclusions

The synthesis of microfiltration membranes from semi-crystalline polymers was investigated, employing spin-casting as an alternative to the extrusion–hot-pressing technique. An in-depth examination of various polymer–filler combinations, with and without stretching, was conducted on membranes designed for the efficient removal of suspended particles. The optimal conditions were identified, striking a crucial balance between pore structure and mechanical integrity.

The feasibility of this approach was demonstrated through a comparative analysis with commercial microfiltration membranes. Stretch-induced thin-film composite membranes for semi-crystalline polymers were evaluated in terms of flow rate and mechanical strength. The environmentally conscious approach, utilizing recycled HDPE, adds an extra layer of significance to the developed membrane synthesis process.

## Figures and Tables

**Table 1:** Compositions involved in membrane preparation.

Membrane	Polymer (mg)		CaCO_3_ (mg)	Stretching
	UHMWPE	HDPE		
M1	700	300	0	Yes
M2	700	300	100	No
M3	700	300	100	Yes
M4	700	300	400	Yes
M5	700	300	700	Yes
M6	700	300	1000	Yes

**Table 2:** Summary of the membranes with filler and stretching ratios.

Membrane	Polymer–CaCO_3_	Uni-Axial Stretch
M1	10:0	1.8
M2	10:1	0
M3	10:1	1.8
M4	10:4	1.8
M5	10:7	1.8
M6	10:10	1.8

## Data Availability

Data are contained within the article.
